# Personal

**Published:** 1861-10

**Authors:** 


### PERSONAL.

Doctor B. Wood, late editor of the *Southern Journal of Medical and Physical Sciences*, Nashville, Tenn., has located in Indianapolis, Ind., where we hope he will meet with the patronage which he merits; he will want no more. The readers of the *Register* are familiar with the name of Dr. Wood. His pen is a pointed one; and we hope it will not forget its old haunts, the pages of the *Register*.

Dr. C. R. Taft, of Mansfield, O., brother of our "T.," is on his way on a visit to Kentucky. He is dressed in *blue*, and ranks as Adjutant of the 15th Ohio Regiment. Go in, Cal., you'll win. W.

